# Effect of robot’s vertical body movement on its perceived emotion: A preliminary study on vertical oscillation and transition

**DOI:** 10.1371/journal.pone.0271789

**Published:** 2022-08-10

**Authors:** Hamed Mahzoon, Ayaka Ueda, Yuichiro Yoshikawa, Hiroshi Ishiguro

**Affiliations:** Intelligent Robotics Laboratory, Department of Systems Innovation, Graduate School of Engineering Science, Osaka University, Osaka, Japan; Public Library of Science, UNITED STATES

## Abstract

The emotion expressions of social robots are some of the most important developments in recent studies on human–robot interactions (HRIs). Several research studies have been conducted to assess effective factors to improve the quality of emotion expression of the robots. In this study, we examined the effects of a robot’s vertical oscillation and transition on the quality of its emotion expression, where the former indicates the periodic up/down movement of the body of the robot, while the latter indicates a one-time up or down movement. Short-term and long-term emotion expressions of the robot were studied independently for the four basic emotions described in the circumplex model of emotions: joy, anger, sadness, and relief. We designed an experiment with an adequate statistical power and minimum sample size of human subjects based on a priori power analysis. Human subjects were asked to evaluate the robot’s emotion expressions by watching its video with/without vertical movement. The results of the experiment showed that for the long-term emotions, the speed of vertical oscillation corresponded to the degree of arousal of the emotion expression as noted in the circumplex model; this indicated that fast oscillations improved the emotion expression with a higher degree of arousal, such as joy and anger, while slow or no oscillations were more suited to emotions with a lower degree of arousal, such as sadness and relief. For the short-term emotions, the direction of the vertical transition corresponded to the degree of valence for most of the expressed emotions, while the speed of vertical oscillation reflected the degree of arousal. The findings of this study can be adopted in the development of conversational robots to enhance their emotion expression.

## Introduction

Emotion expression is one of the most important abilities of social robots in human–robot interactions (HRIs) [1–3]. This ability improves the quality of the robot’s interactions with humans in many aspects [4–7] and is useful even for conveying a robot’s intention to humans [8, 9], e.g., by expressing the robot’s state of mind [10] or establishing more empathy during interactions with people [11]. As an application, it was also shown that emotion expression enabled the robot to play better with children [12, 13], or communicate better with elderly people [14, 15]. However, although efforts to develop expressive social agents have been widely studied [16, 17], designing expressive behaviors that can correctly convey a robot’s emotions in different situations is not simple and these features are difficult to improve because of hardware constraints such as a limited number of joints, facial expression capabilities, and the degree of freedom (DoF) of the social robot.

Previous studies have proposed several methods to improve the emotion expression of a robot in terms of both verbal and non-verbal communications. For verbal communication, several parameters of a robot’s voice and speech, such as the pitch of the voice, the loudness of the speech, or the type of the vocabulary [18] have been reported as effective in expressing a robot’s emotion. It was reported that the emotion of a speaker affected the voice parameter of his/her speech, where parameters such as the pitch, loudness, and prosody of the speech were the most influential ones [19]. To produce an emotional synthetic voice, a mapping model was proposed [20] in which the features of the emotion of a speech manipulated the parameters of the synthesizer, such as articulation, pitch of the voice, and quality of the voice. In another work [21], expressive utterance by an interactive robot was implemented by assembling strings of phonemes with the accents of the pitch. For a tour guide robot [22], the synthetic feature of the robot’s speech was changed based on the emotion of the robot, that is, the robot changed the pitch and voice level of its speech based on its current emotion or change of emotion.

For non-verbal communication, several factors such as the gesture of the robot [23, 24], gaze direction of the robot [25, 26], facial expression [27–30], movement [31–36] and even the color [37, 38] or vibration [39] of the robot were mentioned as effective parameters for improving the robot’s emotion expression. For example, Fiore et al. [25] explored the effectiveness of different types of a robot’s social cues in expressing its emotion, and showed that the proxemic behavior of a robot significantly affects human’s perception about the emotion and social presence of the robot. For a robot with a simple facial expression [28], it was revealed that the recognition of the robot’s facial expression about fear and surprise is difficult, while it is easier for the other basic emotions, i.e., anger, sadness and happiness. The movement of the robot was also mentioned as an effective factor to improve the robot’s emotion expression [31].The speed of the movement, distance to human and the poses of the robot were reported as the important parameters. Additionally, studies about expressing a robot’s emotion and/or naturalness during the robot’s walk or movement have been widely conducted, e.g. determining a suitable set of body joints for expressing different emotions during the walk of a robot [32], realizing the involuntary motion and natural behavior of a robot by utilizing the oscillation of the robot [33], exploring the suitable walk characteristics for the emotion expression based on the stance phase, frequency and the length of footsteps [34], and adopting vertical movement of the robot to express the robot’s emotion during the walk [35].

As mentioned above, several methods, behaviors, and factors have been reported in the literature to represent or improve the emotion expressions of the communicational robots. In this study, we propose two simple factors that can easily enhance the emotion expression of a robot: the vertical oscillation and transition of the body of the robot. Here, the vertical oscillation refers to the periodic up/down movement of the body of the robot, while the vertical transition means a one-time up or down movement of the body of the robot. Since less gestures of communication robots use vertical oscillation and/or transition, adding such a movement to the most of the (previously designed) gestures of the robot seems to be feasible without redesigning and reprogramming the basic gestures. Also, vertical movement and oscillation has been implemented in several studies in order to improve the naturalness of robot’s behavior and impression [29, 31, 32]. Therefore, adding such movements to the gesture of the communication robot seems to be also feasible without damaging the impression of the robot’s behavior.

As a hypothesis, we propose that by adding a vertical oscillation and/or transition to the body of the robot, the quality of the emotion expression represented by the previously designed gesture of the robot can be improved. Since the effective vertical oscillations and/or transitions for different emotions may be different, the study was separately conducted for four basic emotions, i.e., joy, anger, sadness and relief. Also, since short-term and long-term emotion expressions have different characteristics [40], the study was conducted for the short-term and long-term emotion expression of the robot separately. To avoid complexity in the study, we focused on the emotion expression during the conversation of a conversational robot. In this study, the improvement of expression was evaluated subjectively by humans for the *expressivity* and *clarity* of the robot’s expression. Here, expressivity is defined as the extent to which the robot could express its intended emotion, while clarity is defined as the extent to which the emotion of the robot conveyed to them clearly. To verify the proposed hypothesis, an experiment featuring human subjects to evaluate the quality of the emotion expression of the robot with/without the vertical movement was designed and conducted based on a priori power analysis in order to have minimum sample size and enough statistical power. As a preliminary stage of the study, the experiment was conducted utilizing the recorded video of the robot, and the exploration of physical interaction with the robot in a real-world scenario was left for future studies. A statistical hypothesis test was performed on the gathered data and based on the results, the suggested effective vertical movements for each type of emotion were reported. The suggested relation among the speed and/or direction of the vertical movement with the degree of the valence and/or the arousal of the expressed emotion is discussed. Also, the result of the study is compared with a previous report about the characteristics of the body movement of a robot for expressing different emotions [41] based on Laban’s movement analysis [42], and the similarity, difference and the conceivable reasons for such similarity/difference are discussed.

The remainder of this paper is organized as follows. In the section “Materials and method”‎, the general hypothesis of the study as well as the method of this research are explained in detail. In the section “Experiment”‎, the conducted experiments are described. In the sections “Results” and “Discussion”, the results of the experiment and the discussion about the findings of the study are reported. Finally, the conclusion of the study and future works are mentioned in the conclusion section.

## Materials and method

### Hypothesis and experiment design

The aim of this study is to find effective vertical movement(s) that can improve the emotion expression of a conversational humanoid robot. However, for transient emotion expressions and long-lasting ones, the gesture as well as the probable vertical movement of the robot seem to be totally different. For the transient one, the gesture and vertical movement of the robot would consist of short-term representations and movements. For the long term, it is expected that the robot will have long-term movements and expressions. From a psychological perspective, it has been reported that emotion and mood are two distinct phenomena, and usually the term affect is used as an umbrella term for them [40]. “While the emotions have a stimulus event, are comparatively intense, short in duration, and have behavioral implications, the mood consists of rather global, undirected, and mostly unconscious background sensations that are more stable than emotions” [43]. Although the definitions, similarities, and differences in terms such as emotion, feeling, mood, and affect have been widely discussed in psychology for many years [44], there is still discussion about the precise distinctions between them, especially emotion and mood [45]. In this work, as mentioned above, we focus on the transient/long-lasting feature of the expression, and divided the emotion expression of the robot into two categories: short-term expressions and long-term expressions, and studied them independently. For the short-term emotion expression, the effects of both vertical oscillation and transition were studied, while for the long-term expressions, only the effects of vertical oscillation were considered because the non-periodic feature of the vertical transition does not match with the stable and long-lasting features of the long-term emotion expressions, but it is suitable for transient emotions.

Considering the description above, the hypothesis for the short-term and long-term emotion expression of the robot was set as follows: the vertical movement of the robot improves its emotion expression; for the short-term expression, the vertical movement was (the combination of) the vertical oscillation and the vertical transition of the robot as the two independent variables of the study, while for the long-term expression, only the vertical oscillation of the robot was considered. As a measure of the improvement of emotion expression, that is, the dependent variables of the study, the improvement of the expressivity as well as the clarity of the expression were considered, which was defined in the previous section (also see subsection “Apparatus, subjects, and procedure” in section “Experiment” for details about how to measure them in practice). To verify this hypothesis, an experiment containing human subjects was designed with a randomized control-group pretest-posttest design utilizing the analysis method of ANOVA on the gain scores [46], in which each subject firstly evaluates the expressivity and clarity of the emotion expression of the gesture of the robot without the vertical movement as the pretest score of the experiment, and after that evaluates same gesture with a vertical movement as the post-test score of the experiment; the difference of these scores are treated as the gain scores and utilized in the statistical analysis as the dependent variables of the study. Note that the randomization of the order of the videos, in terms of bringing the video of the no-vertical movement to the second order, was not considered in order to prevent probable concern/confusion of the participants about the stop of the vertical movement of the robot in the second video; they may concern if the robot was broken, or focus too much on the eliminated vertical movements but not on the evaluation of the gesture. For the post-hoc multiple comparison, Tukey’s HSD test was considered in this study. See section “Experiment” for the details of the experiment. All the participant agreed with the written consent form approved by the ethics committee for research involving human subjects at the Graduate School of Engineering Science, Osaka University. The consent for the underaged participants were also obtained from their parents/guardians.

### Basic emotions of the robot

**Fig 1:**
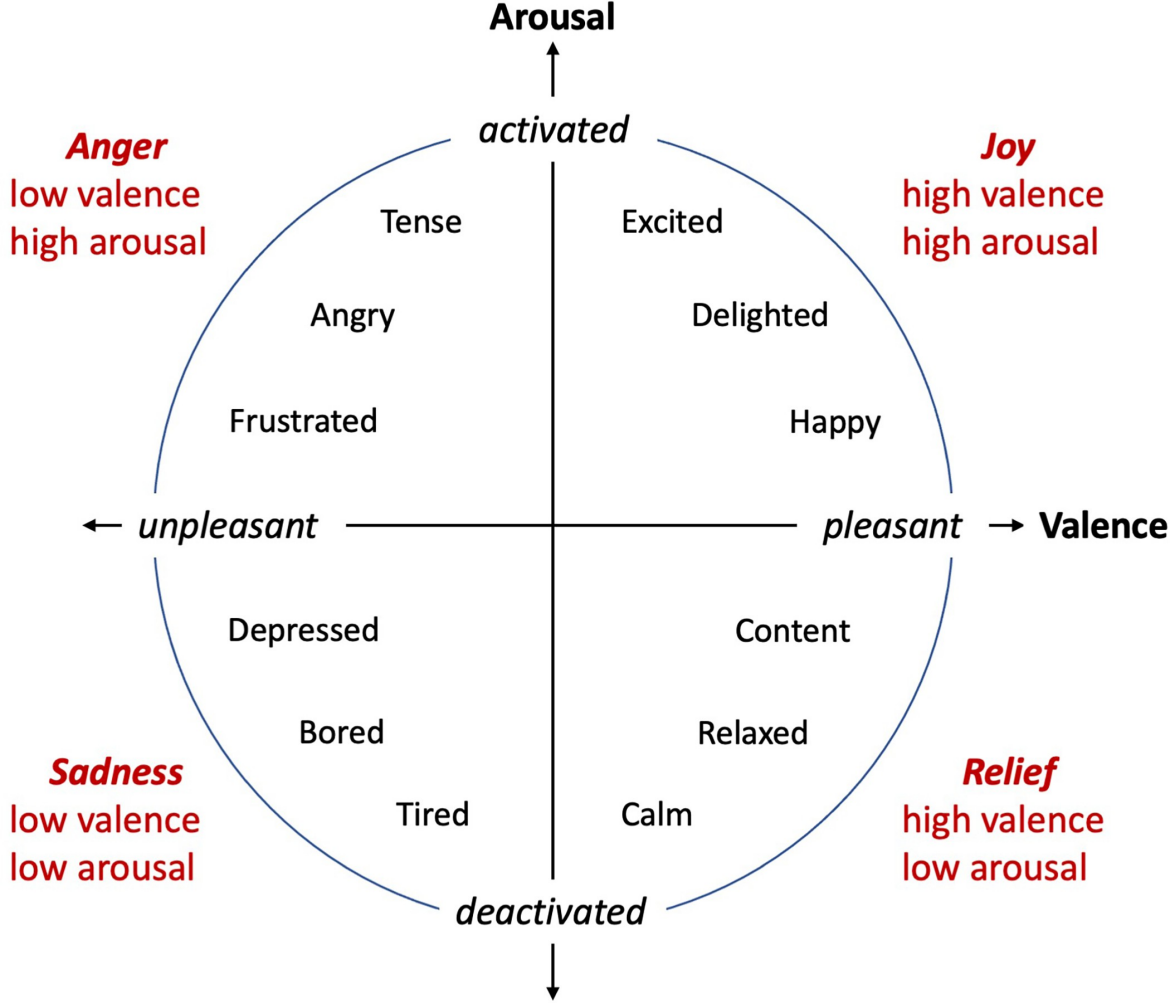
Model of emotion. Circumplex model of emotion [47] utilized as the model of emotions for the robot.

To determine the basic emotions for the study, the circumplex model of emotions [47] was considered as the model describing the robot’s emotions (Fig 1). This model explains the emotions based on two independent factors: 1) the degree of valence and 2) the degree of arousal. Because the model is divided into four areas based on the degree of each factor, namely high/low degree for valence/arousal, a representative emotion that seemed to be easy to understand intuitively by both the designers of the robot’s behavior as well as the participants of the experiment was chosen for each area. In this study, joy (high degree of valence and arousal), anger (low degree of valence but high degree of arousal), sadness (low degree of valence and arousal), and relief (high degree of valence but low degree of arousal) were utilized for the representative emotion of each area.

### Adopted robot and its behavior

#### Robot specifications

**Fig 2:**
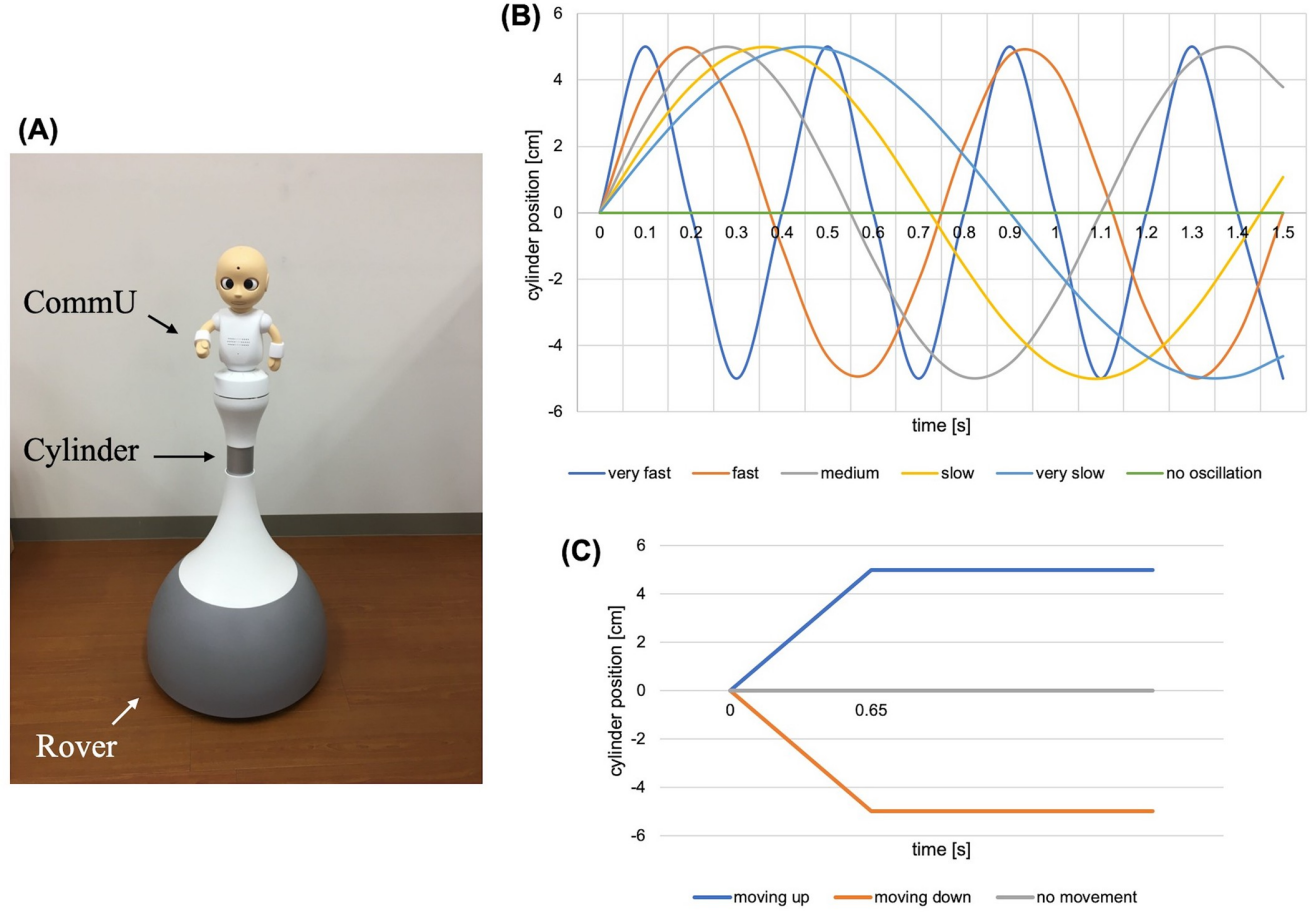
Robot and its vertical oscillation/transition. (A) Robot utilized in the study (Locomotive-CommU), (B) Six different oscillations designed for vertical oscillation of the cylinder of the robot, (C) Three different movements implemented for vertical transition of the cylinder of the robot.

A humanoid robot named locomotive-CommU was used in this study (Fig 2A). This robot consists of a mecanum rover at the bottom, a humanoid robot CommU on the top, and a vertical cylinder connecting these two components. The mecanum rover has four mecanum wheels and is able to move forward, backward, and rotate without changing its position, that is, without moving back and forth. The humanoid robot CommU is a small on-table robot with a cute appearance, which has 14 degrees of freedom (DoF); 2 DoF for its waist, 2 for each arm, 3 for its neck, 3 for the eyeball, 1 DoF for its eyelid, and 1 for its mouth. The vertical cylinder of locomotive-CommU can move up, down, and oscillate. The range of the movement of the cylinder is 30cm, where the highest and the lowest vertical heights of the robot are 1.3m and 1m, respectively.

#### Robot vertical movements for emotion expression

**Fig 3:**
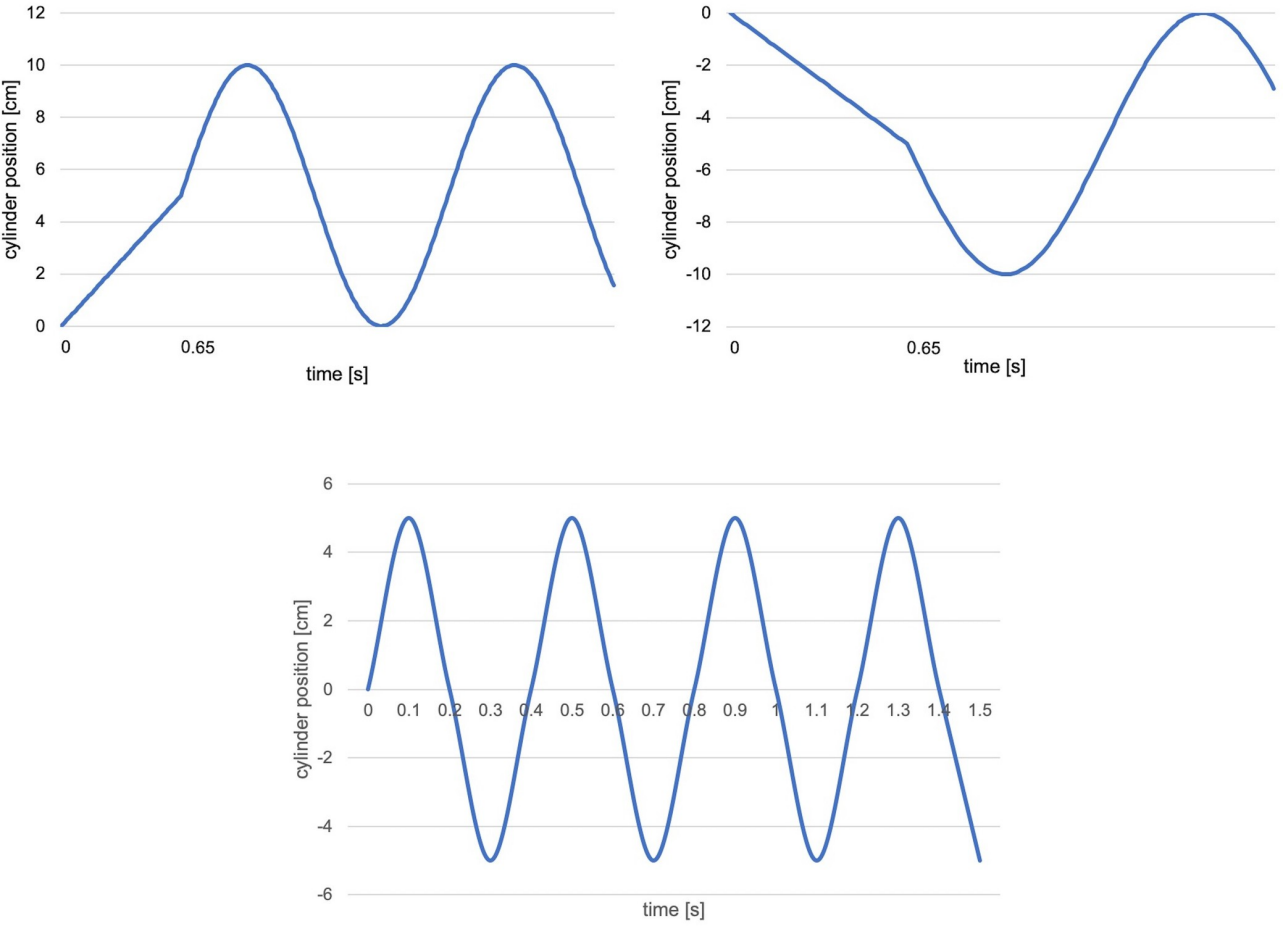
Implemented vertical movements. Examples of the vertical movements of the robot by combining the vertical oscillation and transition of the cylinder.

The vertical movement of the robot was composed of two elements: vertical oscillation and vertical transition of the robot’s cylinder. For the vertical oscillation, six different oscillation speeds for the cylinder including no oscillation were implemented (see Fig 2B). For the vertical transition, changing the height of the cylinder was adopted. It consisted of 3 types: moving up, moving down, and not changing the height (see Fig 2C). Then, the vertical movement of the robot was realized by combining the vertical oscillation and transition. To avoid unnatural vertical movement of the robot, when both the oscillation and transition were adopted, the robot did not start oscillating until the vertical transition was complete (see Fig 3 for examples of the movement). Note that since the utilized robot was ROS compatible including its vertical cylinder, the vertical movements were simply realized by sending the standard ROS locomotion message type, i.e., geometry_msgs/Twist, with the signaling features shown in the figures.

#### Robot gestures for emotion expression

In order to study the effect of a robot’s vertical movement on the improvement of its emotion expression, basic gestures expressing the robot’s emotions are required, so that the vertical movement could be added to them and the improvement of the expression could be studied. Note that in order to avoid complexity in the study, the utterance of the robot was not considered or utilized and left for future works, although it does seem to be another important factor to improve the expression of the robot’s emotions. To determine such gestures for each emotion of the study, we conducted a robot gesture contest in our laboratory. Three different researchers designed their best gesture for each emotion of the robot. Since short-term and long-term emotion expressions were considered in the study, each researcher designed a total of 8 gestures (short-term and long-term emotion expression for each of the 4 emotions, joy, anger, sadness, and relief). Then, the students of the laboratory were asked to compare the video of these gestures and choose the best one for each emotion.

**Fig 4:**
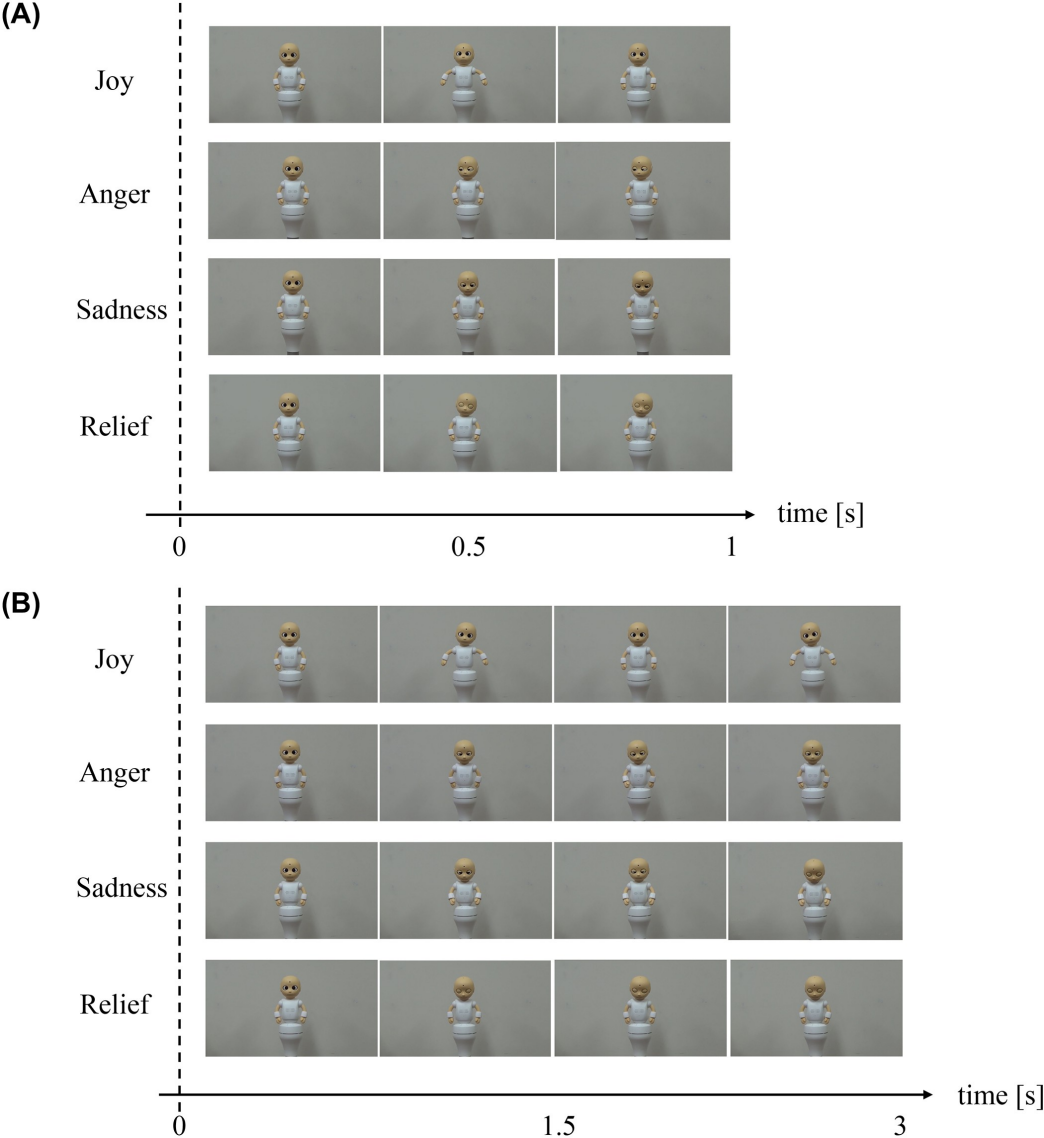
Robot’s gesture for emotion expression. Gestures elected for emotion expression of the robot for (A) short-term expressions, and (B) long-term emotions. The mentioned times in the time axis are the approximate ones and varies depends on the design of each gesture.

Considering 3 different gestures designed for each 4 emotions, 12 videos were prepared for the contest of short-term emotions and 12 for long-term emotions. For the evaluation, first the participants were asked to watch all 12 videos in a random manner (the counterbalance was considered), and after watching each of them the participant was asked to answer the following question, Q1: “In your opinion, what was the emotion that the robot wanted to express in this video?” The participant had to answer by choosing from one of the 4 emotions. After that, the participant was asked to watch all 3 videos designed by different researchers for each emotion, and answer the following question, Q2: “Which video expressed the emotion X of the robot better?”, where X replaced with the word related to each emotion, i.e., joy, anger, sadness, and relief. The most selected gesture in Q2 by the participants for each emotional expression was chosen as the gesture for expressing that emotion in the main experiment. Note that Q1 was asked in order to check whether the designed emotion was conveyed to the participants correctly by each gesture, and also to deepen the discussion of the result of the study (this will be discussed below). 22 university students (19 males and 3 females) at the age of 21 to 27 watched the video related to the short-term emotions while 21 students (18 males and 3 females) watched the video related to the long-term emotions. Fig 4A and 4B show the gestures elected for the short-term and long-term emotion expressions as the result of the contest, respectively.

The number of the votes for each elected gesture as well as the ratio of the perceived emotion for them were also analyzed to see how the participants perceived the emotion expressed by the elected gestures. The result of the number of the votes was as follows for the elected gestures expressing joy, anger, sadness, and relief, respectively: 68%, 50%, 82%, 46% for the short–term, and 76%, 52%, 48%, 43% for the long–term expressions. This result indicates that some gestures won with relatively large number of votes (e.g., joy and sadness for short-term, and joy for long-term expression) and some by a relatively small margin (e.g., relief for short-term, and sadness and relief for long-term expression). For the elected gestures, the evaluation of the participants about Q1, i.e., their perception about the expressed emotion by the gesture, varied for different emotions: Table 1 shows the summary of the answers. As shown in the table, for the short–term expression of relief, the rate of correct answers, i.e., the ratio that the intended emotion of the designed gesture was conveyed to the participants correctly, was low: only 59% of the participants evaluated the gesture as a representation of relief. While for the other emotions, the rates of correct answers were high, i.e., 100%, 82%, and 77% for sadness, anger, and joy, respectively. As shown in the table, 36% of the participants evaluated the short-term expression of relief as the expression of sadness, and 5% evaluated it as a representation of anger. A similar result was obtained for the long–term expressions, as shown in the lower part of the table. Since more than the half of the participants distinguished the emotion of the elected gestures correctly even for the relief, and considering the limitation of the degree of the freedom of the joints and facial expression of the robot for expressing its emotions, as well as the fact that all three designers of the gestures agreed that designing better gestures with the current DoF would be difficult, the elected gestures for each emotion including the one for the relief were evaluated as the adequate ones to be distinguished by most of the people, and were therefore adopted without change in the main experiment of this study.

**Table 1:** Result of the gesture contest.

		Evaluation of the participants			
		Joy	Anger	Sadness	Relief
Designed gesture (for short–term emotion)	Joy	77%	14%	0%	9%
	Anger	0%	82%	14%	5%
	Sadness	0%	0%	100%	0%
	Relief	0%	5%	36%	59%
Designed gesture (for long–term emotion)	Joy	95%	5%	0%	0%
	Anger	0%	91%	10%	0%
	Sadness	0%	10%	81%	10%
	Relief	0%	0%	48%	52%

Evaluations by participants in the robot contest on gestures designed for emotion expression of the robot.

## Experiment

### Short-term emotions

#### Hypothesis

The hypothesis for the short-term emotions is as follows: vertical oscillation and/or vertical transition of the robot improves subjective evaluation by humans regarding the expressivity and/or clarity of a robot’s emotion expression.

#### Apparatus, subjects, and procedure

The robot mentioned in subsection “Robot specifications”, locomotive-CommU, was utilized in the experiment. The experiment was conducted via crowd sourcing, wherein members were asked to answer an online survey over the internet. The participants were asked to watch the video of the robot expressing emotion and evaluate the quality of the emotion expression. For the short-term emotions, the emotion expression was implemented by combining the gestures elected in the gesture contest (Fig 4A) and the vertical movement of the robot, that is, a combination of vertical oscillation and transition. For the vertical transition as the first independent variable of the experiment, all three patterns mentioned in the previous section were adopted as the level of the variable, that is, moving-up, moving-down, and no vertical transition, whereas for the vertical oscillation as the second independent variable of the experiment, three types of oscillations were utilized, that is, no-oscillation, slow oscillation, and fast oscillation because the other oscillations (very fast, medium, and very slow oscillation, see Fig 2B for detail) did not match the short-term gestures of the robot based on our experience, i.e., the combination of the vertical oscillations with the short-term gestures led to very strange and not-synchronized movements. In order to omit the inclusion of such a negative impression, or in other words the factor of the degree of matching, but focusing only on the exploration of the effect of the oscillation speed, the mentioned oscillations were not considered in this experiment. In conclusion, nine different videos related to the different vertical movements were prepared (combination of three types of vertical transitions and three types of oscillations). The lengths of the videos were approximately 1–2 seconds based on the gesture of the robot.

To determine a suitable sample size for the experiment, a priori power analysis was conducted. The results showed that the suitable sample size was N = 2392 with the following parameters: *α* = 0.05, 1−*β* = 0.8, small effect size for interaction effect *f*^2^ = 0.005 [48], number of groups = 9, *df* = 4. Note that since two independent variables were considered for the experiment and the interaction effect of the variables should be considered, the amount of the effect size in the power analysis was set based on the suggested one for the interaction effects, which was described in detail in previous work [48]. For the same reason, the degree of freedom regarding to the interaction effect was set as the parameter of the a priori power analysis, i.e., *df* = 4. As a result, the closest number to the calculated N that the crowd sourcing practically could gather for the experiment was set for the sample size, that is, N = 2250. Table 2 shows the number of participants for each range of age and gender for the experiment. As mentioned in the table, we tried to have the same distribution of the number of subjects for the age and gender ranges. Table 3 shows the number of subjects gathered for the experiment related to each emotion.

**Table 2:** Plan of the experiment for the number of subjects (short-term emotion).

Age	Male	Female	Sum
Less than 15	45	45	90
15 to 19	180	180	360
20 to 29	180	180	360
30 to 39	180	180	360
40 to 49	180	180	360
50 to 59	180	180	360
More than 60	180	180	360
Sum	1125	1125	2250

Number of the participants planned for the evaluation of each short-term emotion expression of the robot.

**Table 3:** Number of subjects for the experiment (short-term emotion).

	Male	Female	Sum
Joy	1128	1170	2298
Anger	986	961	1947
Sadness	1064	1066	2130
Relief	1088	1188	2276
Sum	4266	4385	8651

Number of participants who attended the experiments on short-term emotion expressions.

**Fig 5:**
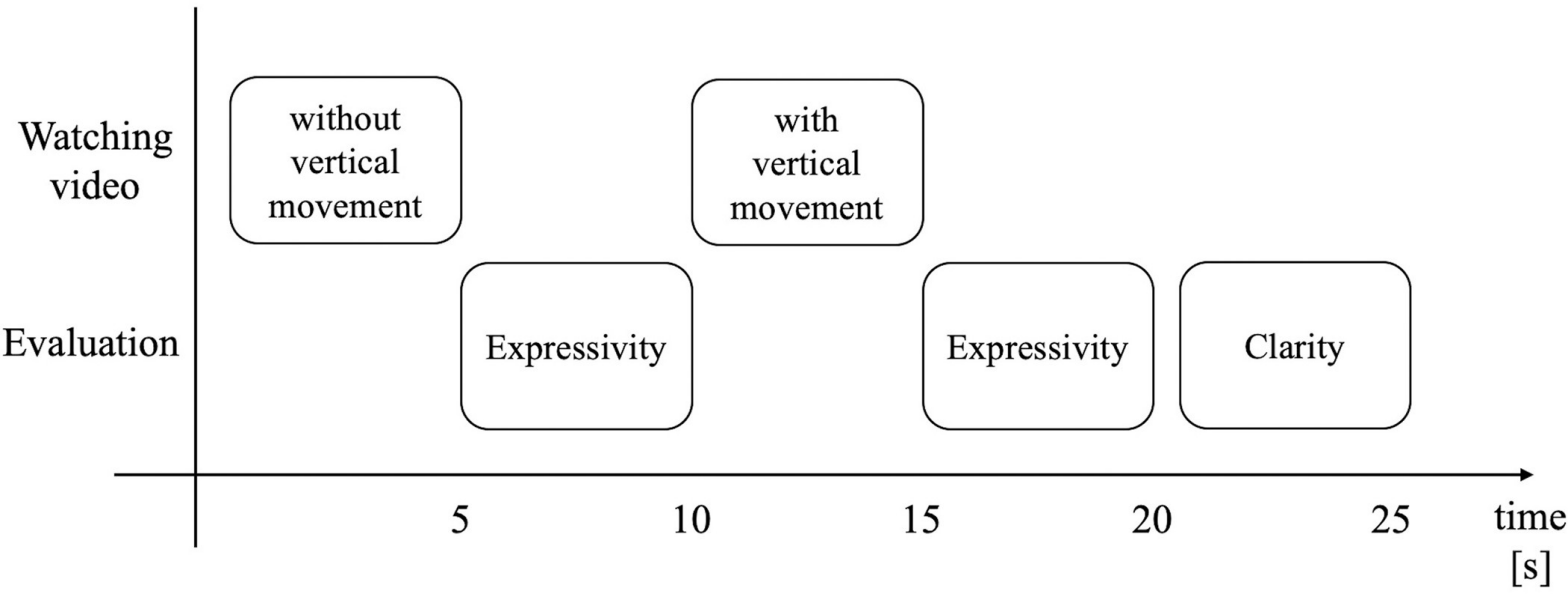
Procedure of the experiment. After watching the first video, the participants evaluated the expressivity of emotions of the robot in the video. Then, they watched the second video and evaluated the expressivity as well as the clarity of emotions of the robot in the video.

Fig 5 shows the procedure of the experiment for each participant. The participants were first asked to watch a video of the robot expressing its emotion with its gesture but without the vertical movement of its cylinder. This gesture was the one elected through the robot gesture contest described in subsection “Robot gestures for emotion expression”. The participants were asked to evaluate the expressivity of the robot’s emotion, that is, the extent to which the robot could express its emotion, by answering question q1: In this video, the robot expressed its emotion X. How much do you think the robot could express it?, where X was replaced with one of the following words depending on the emotion that was programmed for the robot in the video: joy, anger, sadness, or relief. The questions were answered using a 7-level Likert scale, where 7 meant “could express very well” and 1 meant “could not express at all”. Thereafter, the participants watched the video of the robot expressing the same emotion with the same gesture but with the vertical movement. Then the participants were asked to reevaluate the expressivity using the same question, namely q2. Further, the participants were asked to evaluate the improvement in the clarity of the emotion expression of the robot, that is, how much the emotion of the robot was conveyed to the participant, by answering question q3: Compared to the previous video, was the emotion X of the robot conveyed to you more clearly?, where one of the words joy, anger, sadness, or relief replaced the term X. The questions were answered using a 7-level Likert scale, where 7 meant “conveyed very much” and 1 meant “did not convey at all”. The improvement of expressivity, that is, the differences between the scores for q2 and q1, were utilized for the first dependent variable of the experiment, and the improvement of clarity, that is, scores for q3, were used as the second dependent variable of the experiment. As briefly mentioned in subsection “Hypothesis and experiment design”, these dependent variables were utilized as the score gain of the statistical analysis, while the design of the experiment was a randomized control-group pretest-posttest design. In this experiment, the two independent variables (vertical oscillation and transition) were treated as between-subject factors, therefore each participant watched only one video with vertical movement in the post-test stage. The subjects were almost equally distributed to watch one of the nine videos of vertical motion of the robot.

### Long-term emotions

#### Hypothesis

The hypothesis for long-term emotions is as follows: vertical oscillation of the robot improves subjective evaluation by humans regarding the expressivity and/or clarity of a robot’s emotion expression.

#### Apparatus, subjects, and procedur

For the long-term emotions, the same robot, experiment procedure, and evaluation method as those of the short-term emotions were utilized. However, as mentioned before, the vertical transition was not utilized for long-term emotions, and only the vertical oscillation was considered as the independent variable of the experiment. For that, four types of oscillations were adopted: very slow, mid-speed, very fast, and no oscillations (see Fig 2B for details). The other oscillation speeds, that is, slow and fast oscillations in Fig 2B, were not utilized because they did not match with the gestures of the robot based on our experience (see the explanation for the short-term gestures for a detailed explanation). The lengths of the videos for the long-term emotions were 3 to 5 seconds, which were approximately equal to the length of the gesture for each emotion expression.

To determine a suitable sample size for the experiment, a priori power analysis was conducted utilizing the following parameters: *α* = 0.05, 1−*β* = 0.8, small effect size for main effect *f*^2^ = 0.01 [49], number of groups = 4, *df* = 3. Note that since only one independent variable was considered for the experiment and the main effect was the only expected effect, the amount of the expected effect size in the power analysis was set based on the suggested one for the main effects, which is described in detail in the previous work [49]. For the same reason, the degree of freedom regarding to the main effect was set as the parameter of the priori power analysis, i.e., *df* = 3. Because a suitable sample size was obtained as N = 1095, we set the number of participants to the closest number to the calculated sample size considering the practically equal number of the participants that the crowd sourcing could gather for all conditions, that was, N = 1120. Table 4 shows the number of participants designed for each range of age and gender. Table 5 shows the number of subjects who attended the experiments related to each emotion. The procedure of the experiment was the same as that explained for the short-term emotions in the previous section and Fig 5. The only difference was that the adopted gestures for the robot (gestures elected for long-term emotions were utilized instead of those examined in the short-term emotions) and the number of the videos for each emotion (four videos per long-term emotion).

**Table 4:** Plan of the experiment for the number of subjects (long-term emotion).

Age	Male	Female	Sum
Less than 15	8	8	16
15 to 19	92	92	184
20 to 29	92	92	184
30 to 39	92	92	184
40 to 49	92	92	184
50 to 59	92	92	184
More than 60	92	92	184
Sum	560	560	1120

Number of the participants planned for the evaluations of long-term emotion expressions of the robot.

**Table 5:** Number of subjects for the experiment (long-term emotion).

	Male	Female	Sum
Joy	586	601	1187
Anger	598	602	1200
Sadness	592	616	1208
Relief	600	614	1214
Sum	2376	2433	4809

Number of participants who attended the experiments on long-term emotion expressions.

## Results

As noted in the previous sections, for the short-term emotions, two-way ANOVA was conducted to evaluate the effects of two factors (vertical oscillation and vertical transition of the robot) on the improvement of the robot’s short-term emotion expression, that is, the improvement of expressivity and clarity (see subsection “Apparatus, subjects, and procedure” in section “Experiment” for the precise definitions). For the long-term emotions, one-way ANOVA was conducted to evaluate the effect of vertical oscillation of the robot on the improvement. For the post-hoc multiple comparison, Tukey’s HSD test was used. Also, the observed posterior power of the tests (mentioned with 1−*β*) and the effect size of the comparisons in terms of Cohen’s d (mentioned with d) were calculated for the statistical tests. The alpha level of the statistical significance of the tests was set to 0.05 and IBM SPSS Statistics Version 24.0 was used for the analysis. In this section, the results of the statistical test for the short-term and long-term emotions are reported for each of the emotions expressed by the robot, that is, joy, anger, sadness, and relief.

### Short-term emotions

#### Joy

**Fig 6:**
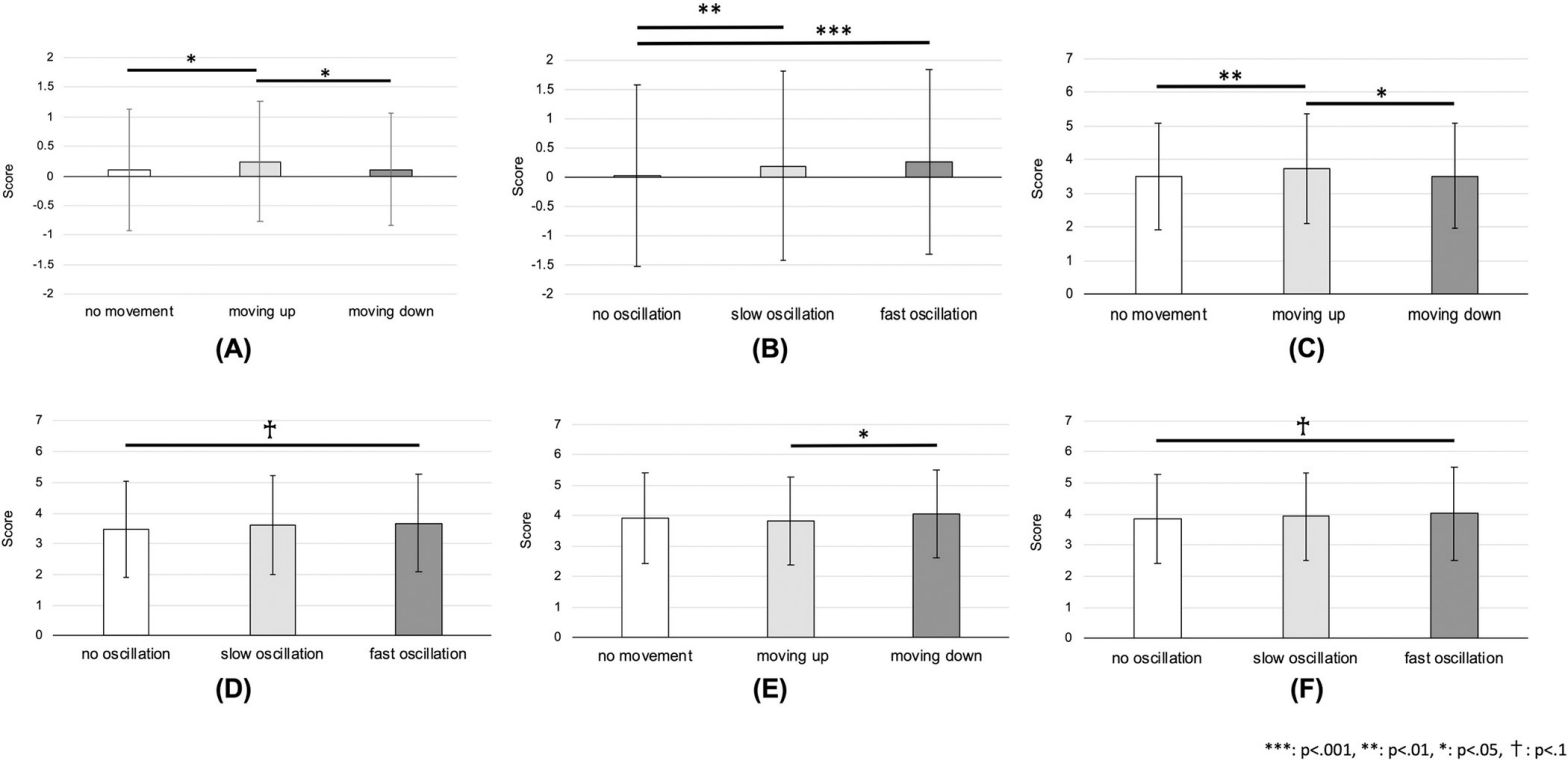
Results for joy and anger in short-term expressions. Score of the expressivity and clarity by different vertical transitions or oscillations of the robot in the case of the short-term representation of joy and anger: **(A)** expressivity of joy by vertical transition and **(B)** by vertical oscillation, **(C)** clarity of joy by vertical transition and **(D)** by vertical oscillation, **(E)** clarity of anger by vertical transition and **(F)** by vertical oscillation. Note that for the expressivity, the improvement of the score is shown as explained in the paper.

For expressivity, the interaction effect was not significant (F(4,2289) = 1.45, p = .213). However, the main effect of the vertical transition as well as the vertical oscillation was significant (F(2,2289) = 5.10, p = .006, 1−*β* = .823 and F(2,2289) = 11.74, p < .001, 1−*β* = .994, respectively). The results of post-hoc comparison showed that the moving-up behavior had a higher score (M = 0.24, SD = 1.01) than the moving-down behavior (M = 0.11, SD = 0.95) as well as the no-movement behavior (M = 0.01, SD = 1.03), where p = .023, d = 0.13 and p = .012, d = 0.14, respectively (see Fig 6A). Additionally, fast and slow oscillations had higher scores (M = 0.25, SD = 1.05 and M = 0.18, SD = 1.07, respectively) compared to the no-oscillation condition (M = 0.01, SD = 0.87), where p < .001, d = 0.24 and p = .002, d = 0.18, respectively (see Fig 6B).

For clarity, the interaction effect was not confirmed (F(4,2289) = 1.21, p = .306). However, the main effect of the vertical transition was significant (F(2,2289) = 5.61, p = .004, 1−*β* = .859), while it was marginally significant for the vertical oscillation (F(2,2289) = 2.91, p = .055, 1−*β* = .570). The post-hoc comparison showed that the moving-up behavior had a higher score (M = 3.74, SD = 1.61) than the moving-down behavior (M = 3.52, SD = 1.57) and no-movement behavior (M = 3.49, SD = 1.58), where p = .017, d = 0.17 and p = .008, d = 0.20, respectively (see Fig 6C). Furthermore, the fast oscillation had a marginally significant higher score (M = 3.48, SD = 1.56) compared to the no-oscillation condition (M = 3.67, SD = 1.58), where p = .055, d = 0.15 (see Fig 6D).

#### Anger

For expressivity, neither the interaction effect nor the main effect was significant (F(4,1938) = 0.547, p = .701 and F(2,1938) < 1.52, p>.220, respectively). For clarity, no interaction effect was found (F(4,1938) = 0.96, p = .431). However, the main effect of the vertical transition was significant (F(2,1938) = 4.72, p = .009, 1−*β* = .791), while it was marginally significant for the vertical oscillation (F(2,1938) = 2.36, p = .095, 1−*β* = .479). The results of post-hoc comparison showed that the moving-down behavior had a higher score (M = 4.05, SD = 1.43) than the moving-up behavior (M = 3.82, SD = 1.43), where p = .001, d = 0.19 (see Fig 6E). Moreover, the fast oscillation had a marginally significant higher score (M = 4.01, SD = 1.49) than the no-oscillation condition (M = 3.84, SD = 1.44), where p = .085, d = 0.14 (see Fig 6F).

#### Sadness

**Fig 7:**
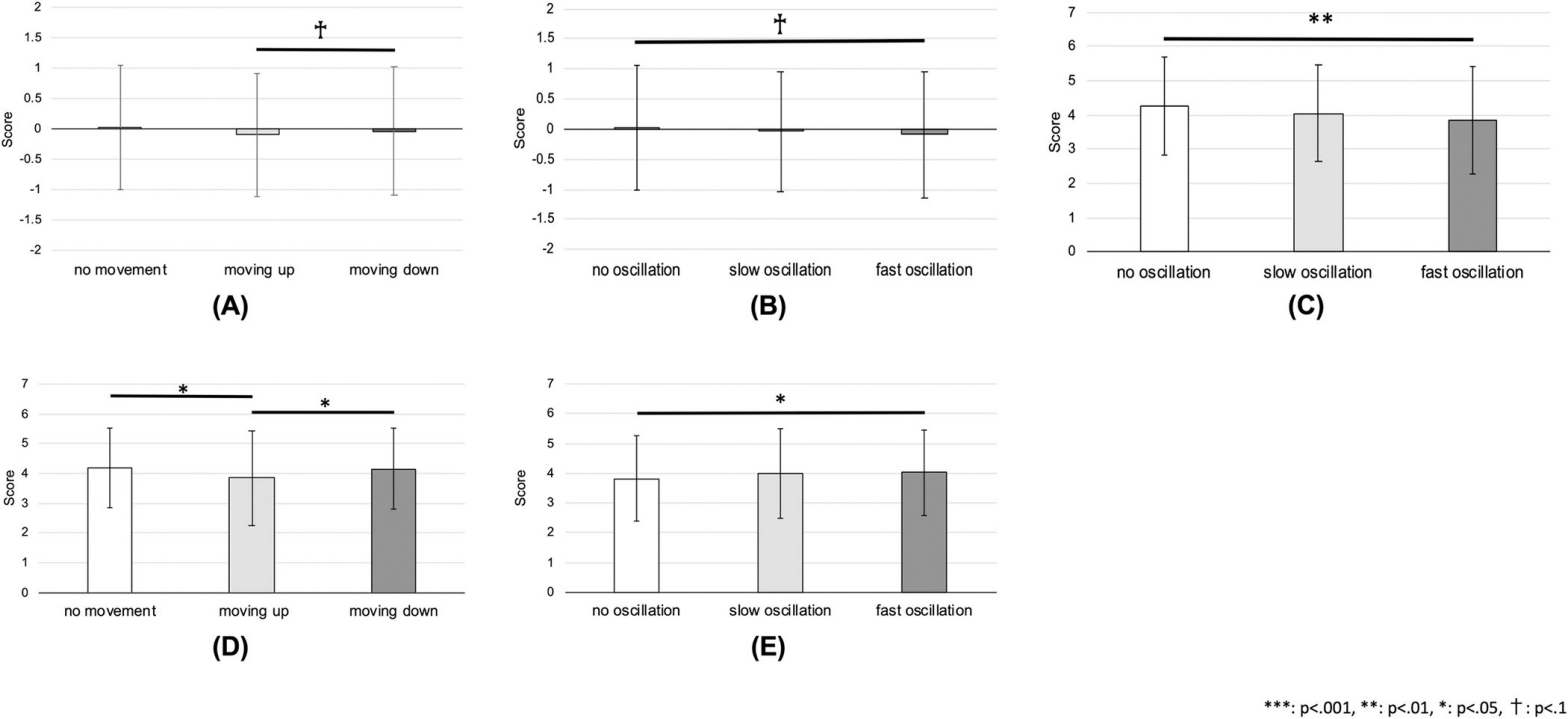
Results for sadness and relief in short-term expressions. Score of the expressivity and clarity by different vertical transitions or oscillations of the robot in the case of the short-term representation of sadness and relief: **(A)** expressivity of sadness by vertical transition and **(B)** by vertical oscillation, **(C)** clarity of sadness by vertical oscillation when the robot had a moving-up behavior and **(D)** by vertical transition when the robot had a fast oscillation, **(E)** clarity of relief by vertical transition. Note that for the expressivity, the improvement of the score is shown as explained in the paper.

For expressivity, no interaction effect was confirmed (F(4,2121) = 0.881, p = .475). However, the main effects of the vertical transition and oscillation were marginally significant (F(2,2121) = 2.85, p = .058, 1−*β* = .560 and F(2,2121) = 2.78, p = .062, 1−*β* = .549, respectively). The results of the post-hoc comparison showed that no-movement had a marginally significant higher score (M = 0.03, SD = 1.02) compared to the moving-up behavior (M = -0.10, SD = 1.01), where p = .055, d = 0.12 (see Fig 7A). Further, no oscillation had a marginally significant higher score (M = 0.03, SD = 1.04) than fast oscillation (M = -0.09, SD = 1.04), where p = .052, d = 0.13 (see Fig 7B).

For clarity, the interaction effect was significant (F(4,2121) = 2.51, p = .040, 1−*β* = .717). An analysis of the following simple main effects showed that the moving-up behavior and fast oscillation had significant effects (F(2,2121) = 4.78, p = .009, 1−*β* = .796, and F(2,2121) = 4.02, p = .018, 1−*β* = .719, respectively). In the case of moving-up behavior, it was revealed that the no-oscillation condition had a higher score (M = 4.25, SD = 1.44) compared to the fast oscillation condition (M = 3.84, SD = 1.58), where p < .001, d = 0.33 (see Fig 7C). In the case of fast oscillation, it was confirmed that the moving-down and no-movement behaviors had higher scores (M = 4.16, SD = 1.38 and M = 4.17, SD = 1.34, respectively) compared to the moving-up behavior (M = 3.84, SD = 1.58), where p = .046, d = 0.26 and p = .034, d = 0.27, respectively (see Fig 7D).

#### Relief

For expressivity, neither the interaction effect nor main effects were confirmed (F(4,2267) = 0.83, p = .507 and F(2,2267)<1.64, p>.194, respectively). For clarity, the interaction effect was not significant (F(4,2267) = 2.30, p = .056). However, the main effect of the oscillation was observed (F(2,2267) = 3.46, p = .032, 1−*β* = .650). The results of the post-hoc comparison showed that the fast oscillation had a higher score (M = 4.02, SD = 1.43) than the no-oscillation condition (M = 3.83, SD = 1.42), where p = .035, d = 0.16 (see Fig 7E). The main effect of vertical transition was not revealed in the experiment (F(2,2267) = 1.39, p = .249).

### Long-term emotions

#### Joy

**Fig 8:**
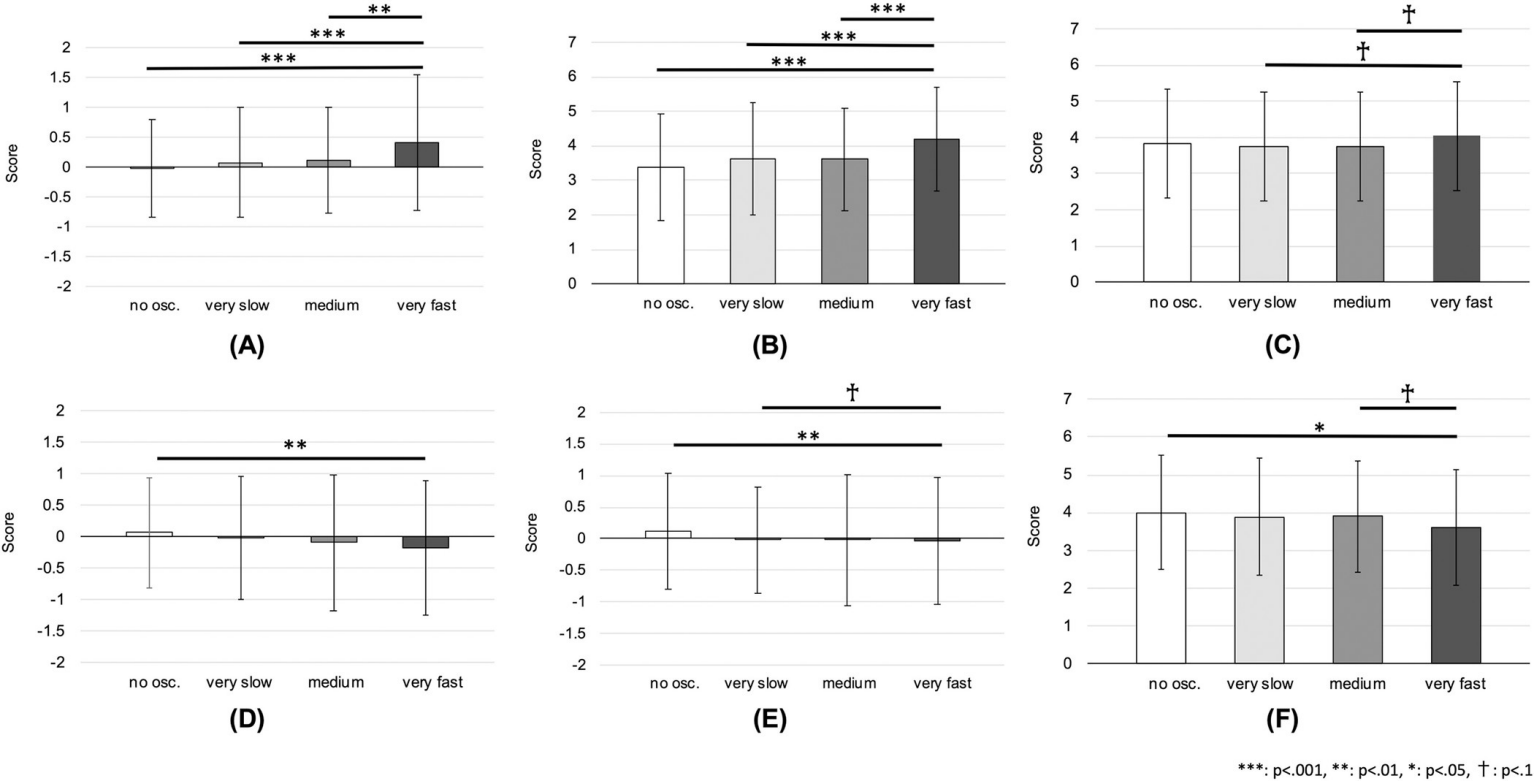
Results for different emotions in long-term expressions. Score of the expressivity and clarity by different vertical oscillations of the robot in the case of the long-term representation of emotions: **(A)** expressivity of joy, **(B)** clarity of joy, **(C)** clarity of anger, **(D)** expressivity of sadness, **(E)** expressivity of relief, **(F)** clarity of relief. Note that for the expressivity, the improvement of the score is shown as explained in the paper.

For expressivity, the main effect of vertical oscillation was significant (F(3,1183) = 11.86, p < .001, 1−*β* = 1.00). Post-hoc analysis showed that the very fast oscillation had a higher score (M = 0.41, SD = 1.14) compared to all other conditions, that is, no oscillation (M = -0.03, SD = 0.81), very slow oscillation (M = 0.08, SD = 0.92), and normal oscillation (M = 0.11, SD = 0.89), where p < .001 for all comparisons, in which d = 0.44, d = 0.33 and d = 0.30, respectively (see Fig 8A).

For clarity, the main effect of vertical oscillation was confirmed (F(3,1183) = 15.17, p < .001, 1−*β* = 1.00). The post-hoc multiple comparison revealed that the very fast oscillation had a higher score (M = 4.19, SD = 1.52) than all other oscillations, that is, no oscillation (M = 3.39, SD = 1.54), very slow oscillation (M = 3.64, SD = 1.62), and normal oscillation (M = 3.61, SD = 1.48), where p < .001 for all comparisons, in which d = 0.65, d = 0.45 and d = 0.48, respectively (see Fig 8B).

#### Anger

For expressivity, the main effect of oscillation was not confirmed (F(3,1196) = 1.27, p = .284). For clarity, the oscillation had a marginally significant effect (F(3,1196) = 2.40, p = .066, 1−*β* = .602). Post-hoc analysis showed that the very fast oscillation had a marginally significant higher score (M = 4.04, SD = 1.49) than the very slow oscillation (M = 3.76, SD = 1.51) as well as normal oscillation (M = 3.75, SD = 1.49), where p = .097, d = 0.23 and p = .090, d = 0.24, respectively (see Fig 8C).

#### Sadness

For expressivity, the oscillation had a significant effect (F(3,1204) = 3.60, p = .013, 1−*β* = .795). The results of the post-hoc multiple comparison test showed that the no oscillation condition had a higher score (M = 0.07, SD = = 0.82) than the very fast oscillation (M = -0.19, SD = 1.06), where p = .009, d = 0.26 (see Fig 8D). For clarity, no significance was found for the main effect of oscillation (F(3,1204) = 0.40, p = .750).

#### Relief

For expressivity, the main effect of the oscillation was significant (F(3,1210) = 5.99, p < .001, 1−*β* = .958). Post-hoc analysis revealed that the no oscillation condition had a higher score (M = 0.12, SD = 0.91) than the very fast oscillation (M = -0.21, SD = 1.01), where p < .001, d = 0.33 (see Fig 8E). In addition, the very slow oscillation had a marginally significant higher score (M = -0.02, SD = 0.84) than the very fast oscillation, where p = .073, d = 0.20 (same figure).

For clarity, the main effect of the oscillation was significant (F(3,1210) = 3.85, p = .009, 1−*β* = .823). The result of the multiple comparison test showed that the no oscillation condition had a higher score (M = 4.00, SD = 1.51) than the very fast oscillation condition (M = 3.61, SD = 1.53), where p = .008, d = 0.32 (see Fig 8F). Additionally, the normal oscillation had a marginally significant higher score (M = 3.91, SD = 1.47) compared to the very fast oscillation, where p = .066, d = 0.25 (same figure).

## Discussion

### Short-term emotions

**Fig 9:**
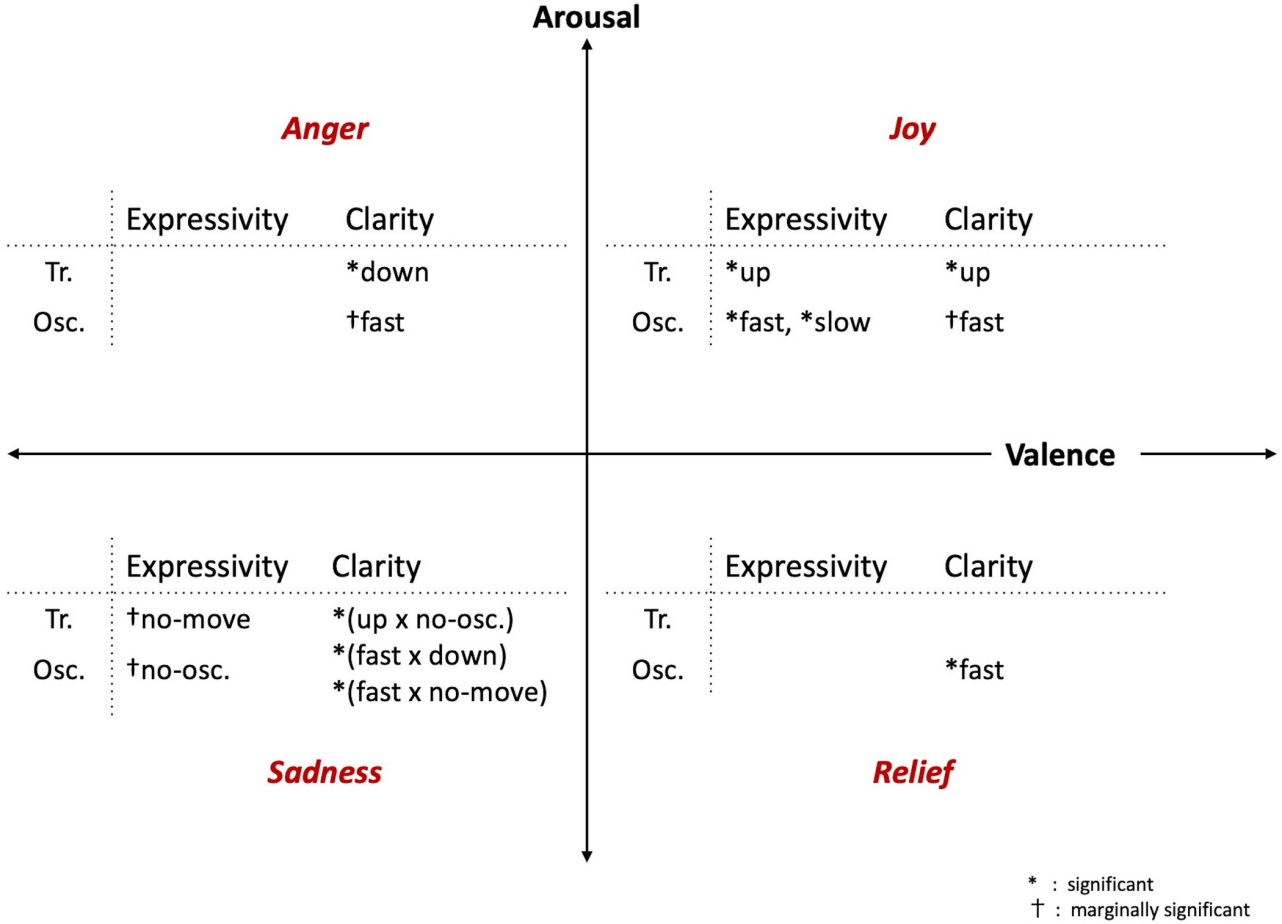
Effective movements for different emotions in short-term expressions. Summary of the effective vertical movements on improvements of expressivity and clarity for short-term emotion expressions of the robot. The vertical transition and oscillation of the robot is denoted with *Tr*. and *Osc*. in the figure, respectively. Note that in the figure, if a movement such as A was effective under the condition B, it is noted as (B × A). Also, the notation *no-osc*. indicates the no-oscillation condition.

Fig 9 shows a summary of the effective vertical transition and oscillation for each short-term emotion of the robot. From this figure, it can be concluded that the vertical transition of the robot corresponded to the degree of valence, while the vertical oscillation corresponded to the degree of arousal for the emotions, except for relief. More precisely, the moving-up behavior was effective in improving the representation of emotion with a high degree of valence, and moving-down behavior was effective in improving the representation of emotions with a low degree of valence, except for relief. Furthermore, having the oscillation was effective in improving the representation of emotions with a high degree of arousal, and having no oscillation was effective for improving the representation of emotion with a low degree of arousal, except for relief. These conclusions were derived from the following facts, which are also summarized in Fig 9.

For joy, i.e., the top-right area of the figure, moving-up behavior was effective for improvement of emotion expression. Moreover, having oscillations (both fast and slow) was effective compared to having no oscillation. For anger, i.e., the top-left area of the figure, moving-down behavior was effective. Further, the fast oscillation had a marginally significant effect on the improvement. For sadness, i.e., the bottom-left area of the figure, no movement was better than moving up or down, while having no oscillation was marginally effective. In addition, in cases where the robot was moving up, having no oscillation improved the expression of sadness. When the robot had fast oscillation, it was better to move down or have no vertical transitions. Finally, for relief, i.e., the bottom-right area of the figure, fast oscillation was effective.

As mentioned above, the only exception to the rule was relief. According to the rule found, the moving-up behavior as well as no-oscillation behavior had to be effective for improvement of the expression of relief. However, while relief has a high degree of valence, there was no effect of the moving-up behavior on the improvement of emotion expression. Further, while relief had a low degree of arousal, there was no effect of the no-oscillation behavior. Instead, the fast oscillation was the effective expression, which is contradictory to the above conclusion. The reason for this seems to be the poor gesture by the robot for expression of relief, as mentioned in the description of Table 1 in subsection “Robot gestures for emotion expression”. Therefore, it appears that in the main experiment, the participants could not distinguish the expression for relief from other emotions such as sadness and anger; thus, the evaluation for adding the vertical movement to the gesture of relief was not similar to those of the other emotions and did not follow the rule found. However, designing a gesture of relief for the robot does not seem to be a simple task, since some previous works reported that humans have a tendency to attach greater weights to negative entities [50], are biased for using negative information [51], and make incorrect judgment of positive faces compared to negative ones [52]. Therefore, for the gestures corresponding to an emotion with a positive valence like relief, more careful designs are required.

Further, the results of this study were compared with those of a previous work [41] in which the relation between the movement of the robot and expressed emotion was analyzed based on the main features of the Laban movement analysis (LMA) [42]. The findings of our study, i.e., the effective factors for improving individual emotion expressions of the robot, were supported by the previous work as follows. In the previous work, the following relationships between improvement of emotion expression of the robot and robot body movement were reported: 1) joy correlates with the straightness of posture, range of body, backward posture, quickness, and powerfulness; 2) anger correlates with the range of body, movement of parts of the body in different directions, quickness, and powerfulness; 3) sadness correlates with low posture, narrowness of body, forward posture, slowness, and weakness; and 4) relief correlates with straightness of posture, backward posture, movement of parts of the body in the same direction, slowness, and weakness. Comparing the effective behaviors revealed in our work with the factors reported in the previous work, the following correspondences could be suggested.

For joy, the moving-up behavior of the robot reported in our work corresponds to the straightness of posture reported in the previous work, since the robot’s height increases via the cylinder’s moving-up motion and consequently provides straightness of posture for the robot. This also corresponds to the range of the body, since the volume of space that the robot occupies increases with the moving-up behavior. The vertical oscillation of the robot, especially the fast movement, is compatible with quickness and powerfulness. For anger, the combination of the moving-down behavior with the looking-left gesture of the robot seems to play the same role as “movement of parts of the body in different directions” reported in the previous work, since the movement of the moving-down behavior is orthogonal with the looking-left gesture of the robot in which the scalar product of these two movement is maximum (for details, see the definition of the space factor in the previous work [53]). Moreover, the fast oscillation of the robot corresponds to quickness and powerfulness.

For sadness, the no-movement behavior of the robot corresponds to narrowness of body, while the moving-down behavior of the robot (in the case where the robot oscillates quickly; see the subsection “Sadness” in the section “Results” ‎for the “Short-term emotions”) is compatible with low posture. Further, the no oscillation behavior corresponds to slowness and weakness. For relief, none of the behaviors of the robot in our study matched with those of the factors mentioned in the previous work. As mentioned before, the poor gestures utilized for the expression of relief by the robot would be a plausible reason for having no corresponding behaviors. Improvement of the gesture for relief may therefore result in finding related behavior between our work and the previous work.

### Long-term emotions

**Fig 10:**
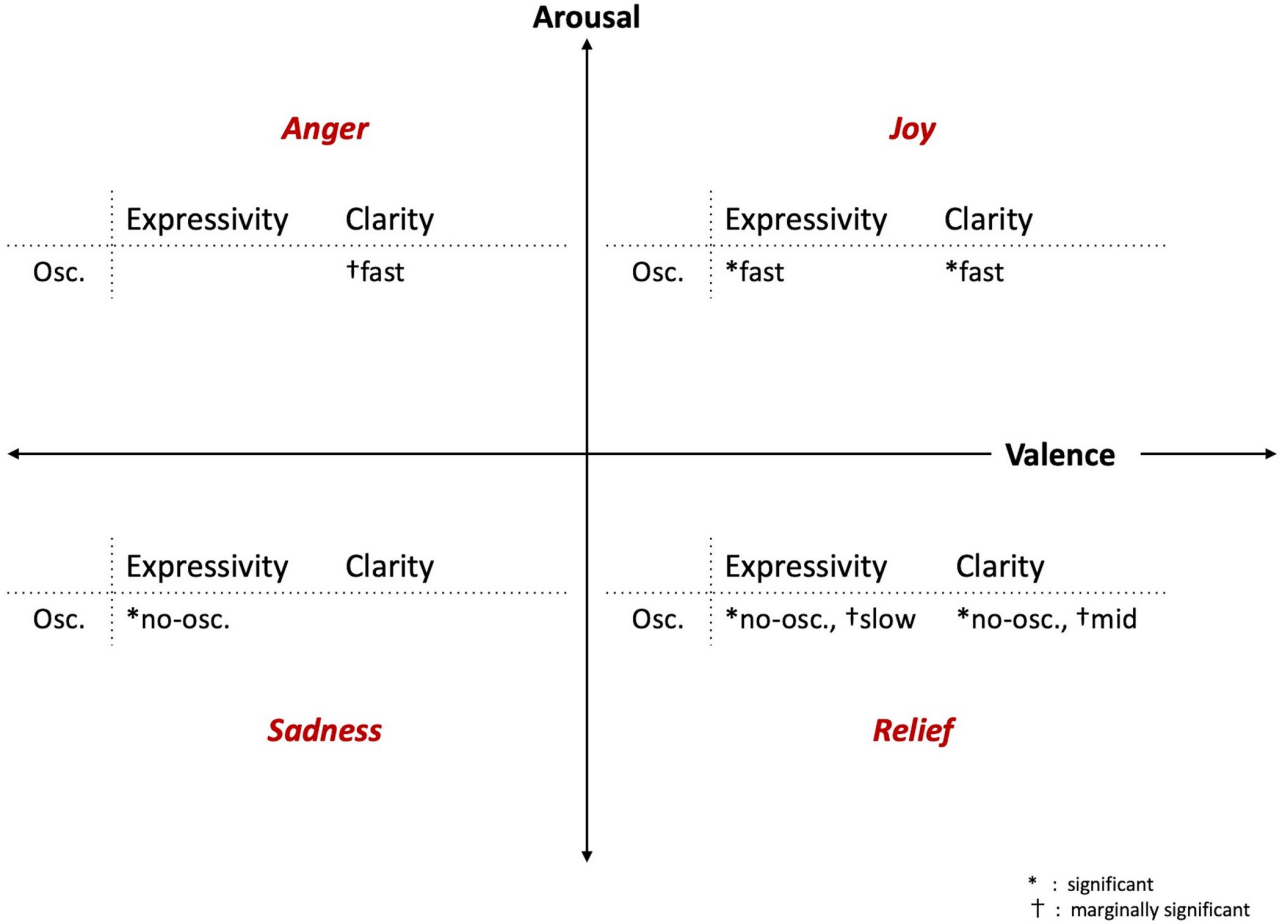
Effective movements for different emotions in long-term expressions. Summary of the effective vertical movements on improvements of expressivity and clarity for long-term emotion expressions of the robot. In the figure, the notation *no-osc*. indicates the no-oscillation condition.

Fig 10 shows a summary of the effective vertical oscillation for each long-term emotion of the robot. For joy and anger, the fast oscillation was effective. For sadness, having no oscillation was effective. Finally, for relief, no oscillation was effective, while the slow and normal oscillations speeds were marginally effective. In the circumplex model of emotions, joy and anger are categorized as emotions with a high degree of arousal, while sadness and relief are classified as having a low degree of arousal. From these results, it is suggested that the speed of the vertical oscillation corresponds to the degree of arousal of emotions in the circumplex model. In other words, the fast vertical oscillation of the robot improves its expression of emotions with high arousal, while the slow or no vertical oscillation enhances expression of low arousal emotions.

### Limitations

Although the effect of the vertical oscillation and transition of the robot on its emotion expression could be summarized based on the significant difference of the mean score of the conditions as shown in Figs 9 and 10, the effect size of the factors was not the same. While the study was conducted with the assumption of small expected effect size as set in the priori power analysis, some factors had a relatively large effect size, e.g., fast oscillation in long–term expression of joy (d = 0.56), while others had a small effect size, e.g., fast oscillation in short–term expression of joy (d = 0.15). Therefore, further studies are required to investigate how the movements of the robot should be improved, especially for the short–term emotions, which had relatively small effect sizes in the result of this study. Also, in the experiment the order of the videos was fixed in order to prevent the probable negative evaluation of the participants about the stopped movement of the robot. However, the extent of such negative effect was not studied objectively and the findings of this study may include the effect of the fixed order of the videos. To solve such limitations, further research would be required to enable a standard psychological experiment with randomized order of the stimuli as well as excluding the probable negative effect.

On the other hand, in the pilot experiment of the study, that is the robot gesture contest held in the laboratory, the number of female participants was quite lower than the number of male participants. In addition, the majors of the all participants were robotics, computer engineering, or computer science. Therefore, the gestures elected for the expression of robot’s emotions might include a strong tendency as a result of such a biased sample, and consequently do not reflect enough the general opinion about robots’ gestures. In future works, conducting such gesture contest using a crowed sourcing study may lead to a more concrete evaluation of a robot’s gesture and the preparation of more precise studies about the effect of robot’s movement on their emotion expression. Also, the cultural context in which the experiment was conducted might play an important role in the selection of the gesture for each emotion. Since it was not controlled in this experiment, a cross-cultural study in future works is necessary in order to reveal details about the way to express the emotions through the body movement of the robot in different cultural contexts. In addition, the gestures were designed by researchers who have no specific specialty in terms of standardized design of the expression, such as facial action coding system (FACS) [54, 55] in the case of face. Systematic and organized design of the gesture and analysis before adopting to the experiment would lead to more precise results and concrete findings in future works.

The other limitation related to the utilized robot was its limited expressive capability. Since the embodiment and human likeness of communicational robots were reported as the important factors for the expressiveness [56–58], gestures created by the simplified humanoid robots (similar to the one used in our experiment) would have limitations. Consequently, the results obtained under such a limitation would have constraints in terms of generalizability: it cannot be extended to the other types of social robots. Therefore, a comparative study on social robots with different levels of expressive capability is required to find out how the same movements may produce a different perception of expression. In this study, a vertical cylinder system was utilized to realize the robot’s vertical movement. However, adopting such a system could be difficult for some communication robots due to their specifications, or even for some interaction scenes. Therefore, study of the vertical movements realized by more common or easy-to-implement systems for the vertical movement and/or oscillation of the robot, such as robot’s torso movement, up-down behavior of the pitch of the robot or the other oscillation generators, can be considered as further possible future studies to evaluate the scalability of the findings of this work.

## Conclusion

In this study, we examined the emotion expression capability of a robot and proposed its vertical oscillation and vertical transition movements as effective factors to improve expressivity and clarity. The expressions of the robot were divided into short-term and long-term emotions and studied independently. Four basic emotions were considered for the robot from the circumplex model of emotions, namely joy, anger, sadness, and relief. To verify the proposed idea, a video experiment was conducted for each emotion, in which human subjects were asked to watch the emotion expression of the robot and subjectively evaluate its expressivity and clarity. Statistical analyses on data gathered from human participants showed that for long-term emotions, the fast oscillation of the robot improved emotion expression with a higher degree of arousal, while slow or no oscillations were better for expressing emotions with a lower degree of arousal. In other words, it was suggested that for long-term emotions, the speed of the vertical oscillation would correspond to the degree of arousal in the circumplex model of the emotion. For the short-term emotions, except for relief, the moving-up transition improved emotion expression with a higher degree of valence, while the moving-down or no vertical transition situations were better for emotions with a lower degree of valence. Thus, it is suggested that that the direction of vertical transition of the robot, or more generally, the degree of expansion of the robot’s body corresponds to the degree of valence in the circumplex model of emotion. Additionally, except for relief, the effect of vertical oscillation of the robot was marginally the same as that for long-term emotions, that is, the speed of vertical oscillation corresponded to the degree of arousal for the emotion. Comparing the findings of this study with the results reported in a previous work [41] in which the relationship between the body movement of the robot and its emotion expression was studied using the main features of the LMA, it is observed that our findings are supported in terms of the effective factors for enhancement of a robot’s emotion expression.

However, to avoid complexity in the study, some of the important modalities of the robot that affect its emotion expressions, such as utterance, voice, color expressions on the cheek, and language were not considered in this work. Studying the effects of a combination of such modalities with the vertical movement, especially language and voice parameters of the robot (which are the most general parameters examined in other social robots), remains as a topic for further research. In addition, the findings of this research were not supported for expression of the relief by the robot. Since the poor gesture for relief was assumed to be the main reason for such failure, adopting more expressive gestures for representation of relief and conducting similar experiments could be considered as avenues for future work. Also, analysis about the effect of age and gender of the participants on the perceived emotion is required to be considered as another essential future work. Finally, conducting real-world human–robot interaction experiments with actual constraints would be an important next step for this study. For a real-word interaction with human, ability of the robot to adapt its behavior based on human’s reaction to prevent the probable short fall in the interaction is one of the essential issues, e.g., producing empathetic response to the emotion of the interlocutor [59]. Research on the human-like interaction skills for the robots to improve emotion recognition and consequently produce adequate response as well as emotional expression is required as another important future work.

## Supporting information

S1 Data (ZIP)

## References

[1] Breazeal C, Brooks R. Robot emotion: A functional perspective. Who needs Emot. 2005;271–310.

[2] Duffy BR. Anthropomorphism and the social robot. Rob Auton Syst. 2003;42(3–4):177–90.

[3] Fong T, Nourbakhsh I, Dautenhahn K. A survey of socially interactive robots. Rob Auton Syst. 2003;42(3–4):143–66.

[4] Fischer K, Jung M, Jensen LC, aus der Wieschen MV. Emotion expression in HRI—when and why. In: 2019 14th ACM/IEEE International Conference on Human-Robot Interaction (HRI). 2019. p. 29–38.

[5] Breazeal C, Dautenhahn K, Kanda T. Social Robotics. In: Siciliano B, Khatib O, editors. Springer Handbook of Robotics. Cham: Springer International Publishing; 2016. p. 1935–72.

[6] Pelikan HRM, Broth M, Keevallik L. “Are you sad, Cozmo?” How humans make sense of a home robot’s emotion displays. In: Proceedings of the 2020 ACM/IEEE International Conference on Human-Robot Interaction. 2020. p. 461–70.

[7] Peña D, Tanaka F. Human Perception of Social Robot’s Emotional States via Facial and Thermal Expressions. ACM Trans Human-Robot Interact. 2020;9(4):1–19.

[8] Nakata T, Sato T, Mori T, Mizoguchi H. Expression of emotion and intention by robot body movement. In: Proceedings of the 5th international conference on autonomous systems. 1998.

[9] Piçarra N, Giger J-C. Predicting intention to work with social robots at anticipation stage: Assessing the role of behavioral desire and anticipated emotions. Comput Human Behav. 2018;86:129–46.

[10] Gockley R, Forlizzi J, Simmons R. Interactions with a moody robot. In: Proceedings of the 1st ACM SIGCHI/SIGART conference on Human-robot interaction. 2006. p. 186–93.

[11] Bates J, others. The role of emotion in believable agents. Commun ACM. 1994;37(7):122–5.

[12] Tielman M, Neerincx M, Meyer J-J, Looije R. Adaptive emotional expression in robot-child interaction. In: 2014 9th ACM/IEEE International Conference on Human-Robot Interaction (HRI). 2014. p. 407–14.

[13] Leite I, Castellano G, Pereira A, Martinho C, Paiva A. Modelling empathic behaviour in a robotic game companion for children: an ethnographic study in real-world settings. In: Proceedings of the seventh annual ACM/IEEE international conference on Human-Robot Interaction. 2012. p. 367–74.

[14] Bemelmans R, Gelderblom GJ, Jonker P, De Witte L. Socially assistive robots in elderly care: A systematic review into effects and effectiveness. J Am Med Dir Assoc. 2012;13(2):114–20. doi: 10.1016/j.jamda.2010.10.002 21450215

[15] Kolling T, Haberstroh J, Kaspar R, Pantel J, Oswald F, Knopf M. Evidence and deployment-based research into care for the elderly using emotional robots. GeroPsych (Bern). 2013.

[16] Cañamero L, Aylett R. Animating Expressive Characters for Social Interaction. Vol. 74. John Benjamins Publishing; 2008.

[17] Cassell J, Sullivan J, Churchill E, Prevost S. Embodied conversational agents. MIT press; 2000.

[18] Severinson-Eklundh K, Green A, Hüttenrauch H. Social and collaborative aspects of interaction with a service robot. Rob Auton Syst. 2003;42(3–4):223–34.

[19] Arnott MI. Towards the simulation of emotion in synthetic speech. J Acoust Soc Speech. 1993;93(2):1097–108. 10.1121/1.405558 8445120

[20] Cahn JE. The generation of affect in synthesized speech. J Am Voice I/O Soc. 1990;8(1):1.

[21] Breazeal C. Emotive qualities in robot speech. In: Proceedings 2001 IEEE/RSJ International Conference on Intelligent Robots and Systems Expanding the Societal Role of Robotics in the the Next Millennium (Cat No 01CH37180). 2001. p. 1388–94.

[22] Nourbakhsh IR, Bobenage J, Grange S, Lutz R, Meyer R, Soto A. An affective mobile robot educator with a full-time job. Artif Intell. 1999;114(1–2):95–124.

[23] Costa S, Soares F, Santos C. Facial expressions and gestures to convey emotions with a humanoid robot. In: International Conference on Social Robotics. 2013. p. 542–51.

[24] Stoeva D, Gelautz M. Body Language in Affective Human-Robot Interaction. In: Companion of the 2020 ACM/IEEE International Conference on Human-Robot Interaction. 2020. p. 606–8.

[25] Fiore SM, Wiltshire TJ, Lobato EJC, Jentsch FG, Huang WH, Axelrod B. Toward understanding social cues and signals in human—robot interaction: effects of robot gaze and proxemic behavior. Front Psychol. 2013;4:859. doi: 10.3389/fpsyg.2013.00859 24348434 PMC3842160

[26] Ruhland K, Peters CE, Andrist S, Badler JB, Badler NI, Gleicher M, et al. A review of eye gaze in virtual agents, social robotics and hci: Behaviour generation, user interaction and perception. In: Computer graphics forum. 2015. p. 299–326.

[27] Kobayashi H, Hara F, Tange A. A basic study on dynamic control of facial expressions for face robot. In: Proceedings of 1994 3rd IEEE International Workshop on Robot and Human Communication. 1994. p. 168–73.

[28] Cañamero L, Fredslund J. I show you how I like you-can you read it in my face?[robotics]. IEEE Trans Syst man, Cybern A Syst humans. 2001;31(5):454–9.

[29] Scheeff M, Pinto J, Rahardja K, Snibbe S, Tow R. Experiences with Sparky, a social robot. In: Socially intelligent agents. Springer; 2002. p. 173–80.

[30] Emotion Breazeal C. and sociable humanoid robots. Int J Hum Comput Stud. 2003;59(1–2):119–55.

[31] Mizoguchi H, Sato T, Takagi K, Nakao M, Hatamura Y. Realization of expressive mobile robot. In: Proceedings of International Conference on Robotics and Automation. 1997. p. 581–6.

[32] Lim H-O, Ishii A, Takanishi A. Basic emotional walking using a biped humanoid robot. In: IEEE SMC’99 Conference Proceedings 1999 IEEE International Conference on Systems, Man, and Cybernetics (Cat No 99CH37028). 1999. p. 954–9.

[33] Miyashita T, Ishiguro H. Human-like natural behavior generation based on involuntary motions for humanoid robots. Rob Auton Syst. 2004;48(4):203–12.

[34] Venture G, Kadone H, Zhang T, Grèzes J, Berthoz A, Hicheur H. Recognizing emotions conveyed by human gait. Int J Soc Robot. 2014;6(4):621–32.

[35] Yagi S, Ise N, Yu S, Nakata Y, Nakamura Y, Ishiguro H. Perception of Emotional Gait-like Motion of Mobile Humanoid Robot Using Vertical Oscillation. In: Companion of the 2020 ACM/IEEE International Conference on Human-Robot Interaction. 2020. p. 529–31.

[36] Mahzoon H, Ogawa K, Yoshikawa Y, Tanaka M, Ogawa K, Miyazaki R, et al. Effect of self-representation of interaction history by the robot on perceptions of mind and positive relationship: a case study on a home-use robot. Adv Robot. 2019;33(21):1112–28.

[37] Sugano S, Ogata T. Emergence of mind in robots for human interface-research methodology and robot model. In: Proceedings of IEEE international conference on robotics and automation. 1996. p. 1191–8.

[38] Terada K, Yamauchi A, Ito A. Artificial emotion expression for a robot by dynamic color change. In: 2012 IEEE RO-MAN: The 21st IEEE International Symposium on Robot and Human Interactive Communication. 2012. p. 314–21.

[39] Song S, Yamada S. Expressing emotions through color, sound, and vibration with an appearance-constrained social robot. In: 2017 12th ACM/IEEE International Conference on Human-Robot Interaction (HRI. 2017. p. 2–11.

[40] Frijda NH, others. Varieties of affect: Emotions and episodes, moods, and sentiments. 1994.

[41] Masuda M, Kato S. Motion rendering system for emotion expression of human form robots based on laban movement analysis. In: 19Th international symposium in robot and human interactive communication. 2010. p. 324–9.

[42] Laban R, Ullmann L. The mastery of movement. 1971.

[43] Ekkekakis P. Affect, mood, and emotion. Meas Sport Exerc Psychol. 2012;321.

[44] Scherer KR. What are emotions? And how can they be measured? Soc Sci Inf. 2005;44(4):695–729.

[45] Beedie C, Terry P, Lane A. Distinctions between emotion and mood. Cogn Emot. 2005;19(6):847–78.

[46] Dimitrov DM, Rumrill PD Jr. Pretest-posttest designs and measurement of change. Work. 2003;20(2):159–65. 12671209

[47] Russell JA. A circumplex model of affect. J Pers Soc Psychol. 1980;39(6):1161.

[48] Hair JF Jr, Hult GTM, Ringle C, Sarstedt M. A primer on partial least squares structural equation modeling (PLS-SEM). Sage publications; 2016.

[49] Cohen J. Statistical power analysis for the behavioral sciences. Academic press; 2013.

[50] Rozin P, Royzman EB. Negativity bias, negativity dominance, and contagion. Personal Soc Psychol Rev. 2001;5(4):296–320.

[51] Vaish A, Grossmann T, Woodward A. Not all emotions are created equal: the negativity bias in social-emotional development. Psychol Bull. 2008;134(3):383. doi: 10.1037/0033-2909.134.3.383 18444702 PMC3652533

[52] Steiner JE. Human facial expressions in response to taste and smell stimulation. In: Advances in child development and behavior. Elsevier; 1979. p. 257–95. 10.1016/s0065-2407(08)60349-3 484324

[53] Masuda M, Kato S, Itoh H. Emotion detection from body motion of human form robot based on laban movement analysis. In: International Conference on Principles and Practice of Multi-Agent Systems. 2009. p. 322–34.

[54] Ekman P, Friesen W V. Facial action coding system. Environ Psychol \& Nonverbal Behav. 1978.

[55] Cohn JF, Ambadar Z, Ekman P. Observer-based measurement of facial expression with the Facial Action Coding System. Handb Emot elicitation Assess. 2007;1(3):203–21.

[56] Wainer J, Feil-Seifer DJ, Shell DA, Mataric MJ. The role of physical embodiment in human-robot interaction. In: ROMAN 2006-The 15th IEEE International Symposium on Robot and Human Interactive Communication. 2006. p. 117–22.

[57] Fink J. Anthropomorphism and human likeness in the design of robots and human-robot interaction. In: International Conference on Social Robotics. 2012. p. 199–208.

[58] Kwak SS, Kim Y, Kim E, Shin C, Cho K. What makes people empathize with an emotional robot?: The impact of agency and physical embodiment on human empathy for a robot. In: 2013 IEEE RO-MAN. 2013. p. 180–5.

[59] Filippini C, Perpetuini D, Cardone D, Merla A. Improving Human—Robot Interaction by Enhancing NAO Robot Awareness of Human Facial Expression. Sensors. 2021;21(19):6438. doi: 10.3390/s21196438 34640758 PMC8512606

